# A rare case of severe tricuspid regurgitation caused by detached stent falling into the right ventricle after transjugular intrahepatic portosystemic shunt

**DOI:** 10.1186/s13019-022-01794-x

**Published:** 2022-03-19

**Authors:** Feng Xiong, Kunyue Tan, Lijian Cheng, Yong Luo, Zhengkai Zhao

**Affiliations:** Department of Cardiology, Cardiovascular Institute of Chengdu, The Third People’s Hospital of Chengdu, No. 82, Qinglong Road, Qingyang District, Chengdu, 610031 Sichuan China

**Keywords:** Transjugular intrahepatic portosystemic shunt (TIPS), Stent falling, Tricuspid, Liver Cirrhosis regurgitation

## Abstract

**Background:**

Transjugular intrahepatic portosystemic shunt (TIPS) is an important method to alleviate cirrhotic portal hypertension. But the falling and fracture of the stent which detaches into the heart is a potentially fatal threat.

**Case presentation:**

We present a case of severe tricuspid regurgitation caused by detached stent falling into the right ventricle after transjugular intrahepatic portosystemic shunt.

**Conclusion:**

Great attention should be paid to the serious complication of stent fracture after TIPS especially when the dual stent technique is used in TIPS.

## Background

TIPS aims to establish an effective blood flow channel between the portal vein and the systemic circulation to relieve portal hypertension. It is an important method for the treatment of refractory ascites and bleeding caused by esophageal and gastric varices. The main complications of stent related to the TIPS stent bending, shortening and displacement. Postoperative stent fracture is rare.

## Case presentation

The patient was 67 years old and male with a history of hepatitis B going back 30^+^ years and a history of hypertension going back 10^+^ years. He had never taken antiviral treatment, but his blood pressure was under control. He had undergone repeated hematemesis 5^+^ years earlier, and gastroscopy revealed severe esophageal varices and gastric fundus varices. Intravenous pumping of proton-pump inhibitor (PPI) and somatostatin and gastroscopic tissue glue injection into the varicose veins showed a poor hemostatic effect. Abdominal CT indicated liver cirrhosis, splenomegaly, a small amount of ascites, and portal hypertension accompanied by collateral circulation. Computed tomography angiography (CTA) showed multiple calcification of the abdominal aortic wall, dilated portal and splenic veins, and varices of the lower esophagus and gastric fundus. Transjugular intrahepatic portosystemic shunt (TIPS) was performed, and 2 stents (1 COOK bare stent, and 1 BARD covered stent) were placed. The discharge diagnosis of the patient 5 years ago was “post-hepatitis B cirrhosis (decompensation stage) with portal hypertension, upper gastrointestinal hemorrhage, post-TIPS, and hypertension grade 3.”

**Fig. 1:**
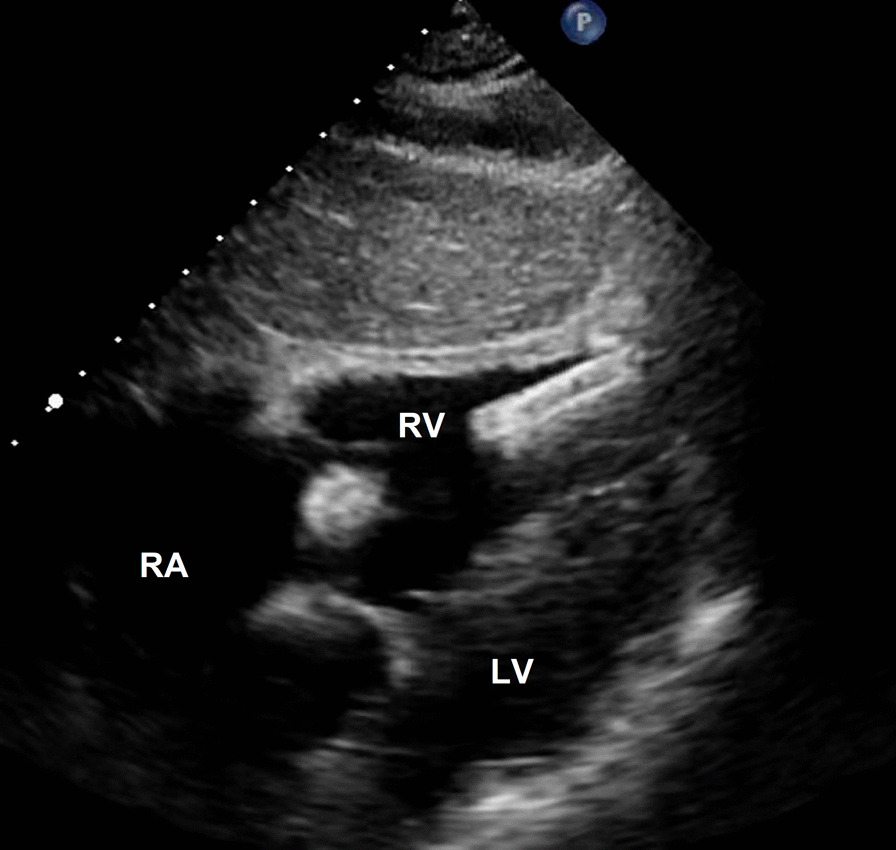
Transthoracic echocardiography shows two hyperechoic tubular shadows at the tricuspid valve orifice and in the right ventricle. LV: left ventricle, RA: right atrium, RV: right ventricle

**Fig. 2:**
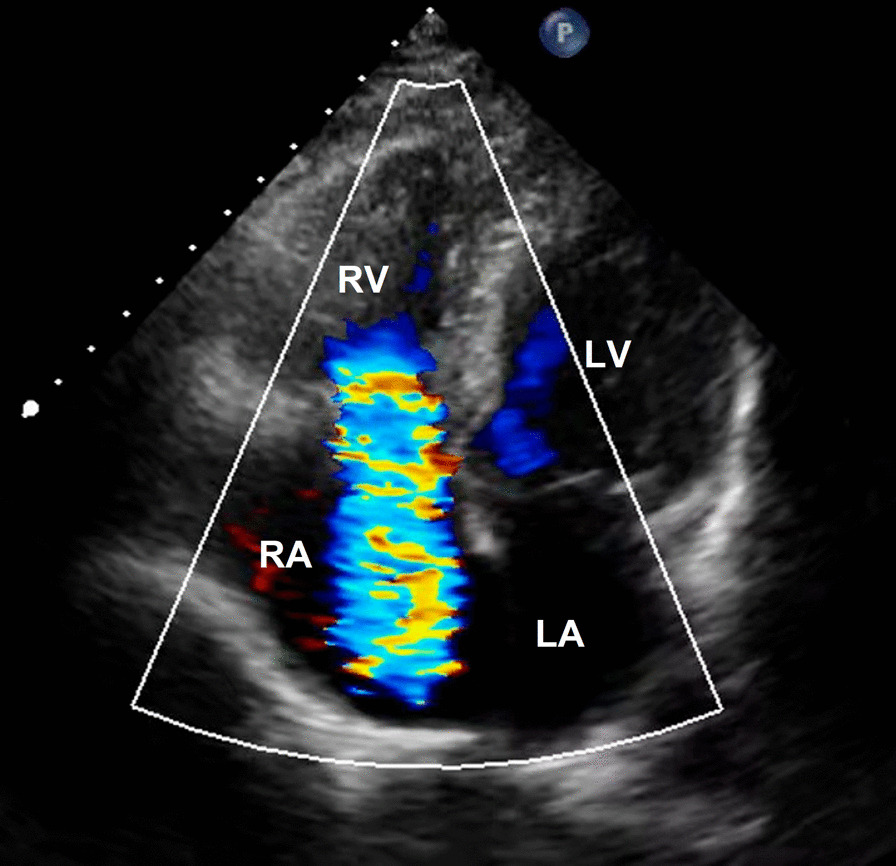
Transthoracic echocardiography shows severe tricuspid regurgitation. LA: Left atrium, LV: left ventricle, RA: right atrium, RV: right ventricle

**Fig. 3:**
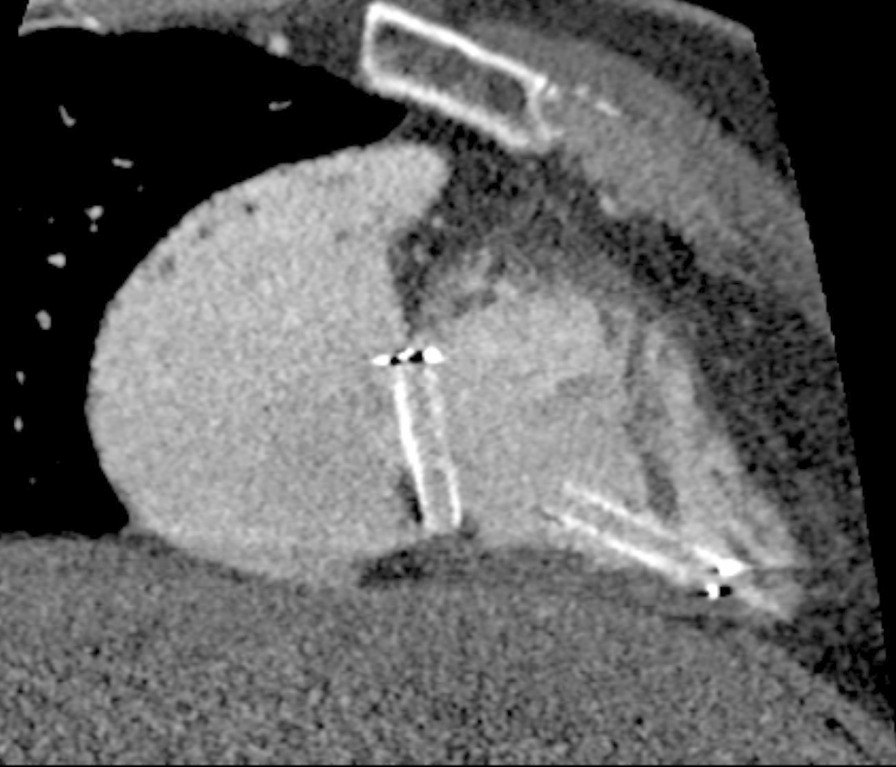
Cardiac CTA shows tubular high-density shadows at the tricuspid valve and in the right ventricle

**Fig. 4:**
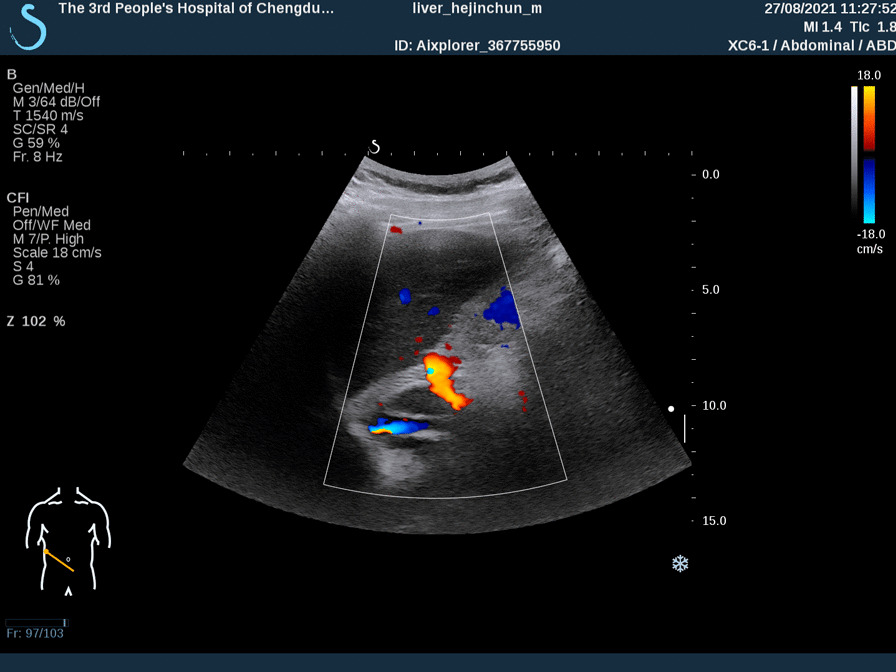
Liver ultrasound shows tubular hyperechoic shadow between the right hepatic vein and the right branch of the portal vein without blood flow signal in it

**Fig. 5:**
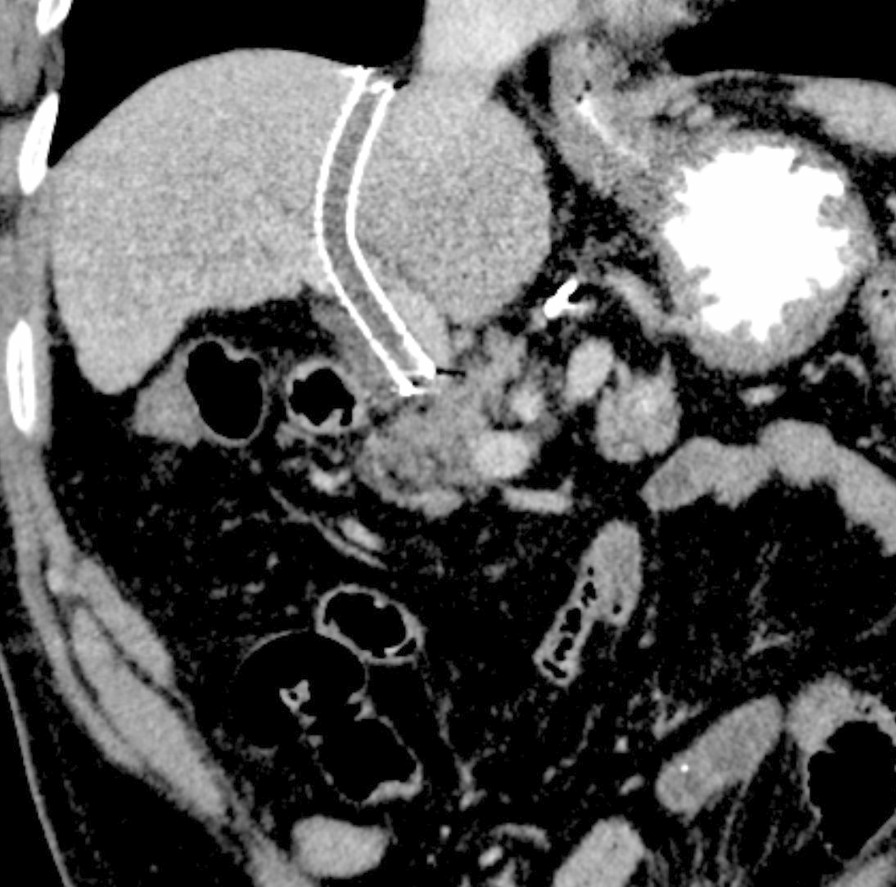
Abdominal CT reconstruction shows tubular high-density shadow in the right branch of the portal vein that passes through the liver parenchyma to the proximal end of the inferior vena cava with low-density shadow in the lumen and no contrast agent filling

**Fig. 6:**
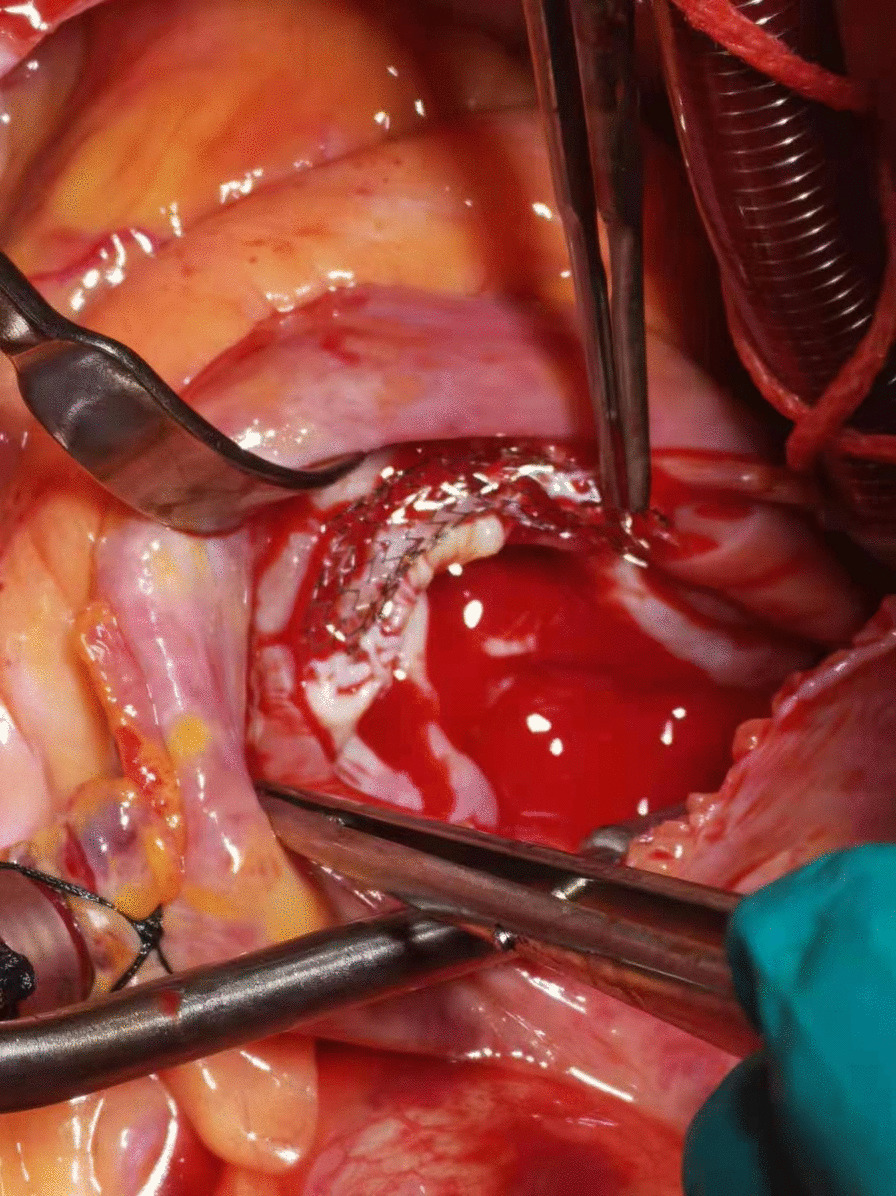
A stent fragment that attached tightly to the posterior leaflet of the tricuspid valve was exposed during surgery

**Fig. 7:**
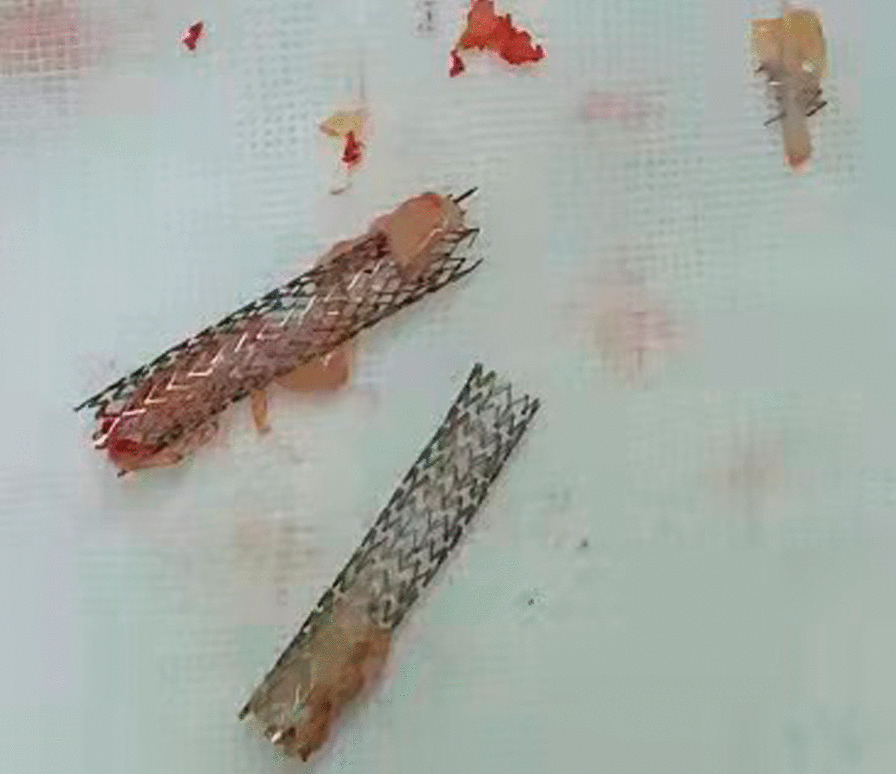
The 3 stent fragments removed by surgery

At a physical examination 10^+^ days ago, a 3/6 grade systolic blowing murmur was detected at the left edge of the sternum, hepatic jugular vein reflux sign was negative, and there was no heart palpitation, shortness of breath, syncope, amaurosis, or lower extremity edema detectable. Cardiac color Doppler ultrasound revealed two tubular hyperechoic shadows in the right ventricle (Fig. 1), significantly enlarged right heart (right atrium with transverse diameter of 56 mm, and right ventricular with base transverse diameter of 51 mm), and severe tricuspid regurgitation (Fig. 2). Cardiac CTA showed tubular high-density shadow in the tricuspid valve area and right ventricle (Fig. 3). Liver ultrasound showed tubular hyperechoic shadow between the right hepatic vein and the right portal vein without blood flow signal in it (Fig. 4). Abdominal CT reconstruction showed tubular shadow in the right portal vein that passes through the liver parenchyma to the proximal end of the inferior vena cava with low-density shadow filling within the tubular structure (Fig. 5). Under general anesthesia, the foreign pieces in the right heart were removed and tricuspid valve replacement surgery was performed. During the operation, a stent fragment was found to be tightly attached to the posterior leaflet of the tricuspid valve (Fig. 6), and the other two fragments of the stent were attached to the right ventricular trabeculae, one of which was short and small. The stent fragment at the valve leaflet attachment location was difficult to remove, so part of the posterior leaflet was cut along the attached stent to completely remove the stent fragment. The hyperplasia tissue at the root of the leaflet where the fragment attached was cleared, and the stent fragments in the right ventricular were also removed, retrieving a total of three stent fragments (Fig. 7). Half a month after the operation, cardiac color Doppler ultrasound showed that the diameter of the right heart had returned to normal and no tricuspid regurgitation was observed.

## Discussion and conclusions

In patients with liver cirrhosis complicated with portal hypertension, continuously elevated portal pressure is the main cause of varices and varix rupture and bleeding. Rupture and bleeding of esophageal and gastric varices is a serious clinical complication of liver cirrhosis, usually accompanied by a large amount of bleeding and high mortality [1]. TIPS surgery aims to establish an effective blood flow channel between the portal vein and the systemic circulation to relieve portal hypertension. As such, it is an important method for the treatment of bleeding caused by esophageal and gastric varices. In recent years, the dedicated Viatorr stent for TIPS has been widely used. However, many hospitals in China and other countries still use the dual stent technique, in which the bare stent and the covered stent are superimposed and released to simulate a Viatorr stent. The Viatorr stent has a two-segment design with circular segmentation marker to assist intraoperative positioning. Its self-expanding nitinol alloy component is more suitable for liver anatomy that it is not prone to bending, shrinking, and capping at both ends of the stent. Therefore, the incidence of shunt narrowing or occlusion is lower than TIPS techniques. In the dual-stent technique, the two stents need to be released separately, and there is no positioning marker, which significantly increases the difficulty of the procedure.

The main complications related to the TIPS operation are biliary hemorrhage, abdominal hemorrhage, and stent displacement. After the operation, serious complications such as shunt malfunction or hepatic encephalopathy may occur. Shunt malfunction is mainly caused by stenosis and occlusion of the shunt. Postoperative stent fracture is extremely rare, and it has been reported only in a few studies worldwide. In 2014, Zabicki et al. [2] reported a case of stent fracture 6 months after TIPS that stent fragments were found in the inferior vena cava, right atrium, and right ventricle. This may be related to liver deformation, diaphragm movement, stent placement angle, and other factors, all of which subject the stent to high tension. The ideal shunt is a straight imaginary channel established between the hepatic vein (right or middle) and the right portal vein. However, in the case reported by Ding et al. [3], the stent fractured without forming a special angle, and it is speculated that the free part of the proximal end of the bare stent was affected by breathing movement and the heart beating, and the superimposed part of the stent, especially the proximal end, was subjected to continuous force. In addition to the above factors, Komaki et al. [4] believed that the fracture of the stent is also related to its own design and material that once the self-expanding nickel-titanium alloy bare stent with open-hole design breaks at the joint. This not only causes the stent to fracture but also causes severe fragmentation of the broken ends.

The intraoperative procedure and postoperative follow-up data of this patient is unknown. However, the patient was in generally good condition after TIPS operation, without hepatic encephalopathy or gastrointestinal hemorrhage. Although the specific time and cause of the stent fracture are unknown, CT reconstruction images taken 5^+^ years after the operation showed that the covered stent in the liver had a large curvature, indicating that the stent was under significant bending force, and the stent was filled with low-density materials suggesting that thrombus formation caused the obstruction of the shunt, confirmed by liver ultrasound showing no blood flow in the stent. The bare stent that had migrated into the right ventricle had broken into three pieces, which were tightly attached to the adjacent myocardial tissue. Among them, the fragment behind the posterior leaflet of the tricuspid valve directly caused dysfunction of the tricuspid valve, resulting in severe regurgitation. Judging from the prominent proliferation of surrounding myocardial tissues stimulated by the stent fragments and the degree of attachment of these pieces, the stent had fractured some time earlier. It is not clear whether the stent broke into three fragments before entering the right ventricle or whether the stent was squeezed and broke into three pieces after the it had completely detached into the right ventricle. However, the mechanical squeezing that occurs in the heart cavity is worth consideration. Although the severe regurgitation of the tricuspid valve had caused a significant enlargement of the right heart, because the right heart usually has a good tolerance to volume load, the patient did not show the expected symptoms.

Not many health care professionals have experience in the treatment of the kind of stent fracture that occurs after TIPS. Toyoda et al. [5] reported a case of a stent migrating into the right ventricle 24 h after a TIPS operation. The patient had chest pain and frequent premature atrial beats, and there was also risk of further movement of the stent. The physicians decided to remove the stent surgically. In most cases, the patients had neither clinical symptoms nor acute hemodynamic disorders, and so they were placed under regular palliative observation. For this patient, the damage to the tricuspid valve by the fractured piece caused severe tricuspid regurgitation and significant enlargement of the right heart, which are clear surgical indications.

Any implanted device that detaches into the heart is a potentially fatal threat. Therefore, surgeons should pay great attention to the serious complication of stent fracture after TIPS especially when the dual stent technique is used in TIPS. The standard operation procedure of TIPS and regular postoperative evaluation of stent shape and shunt function are especially important to the prevention of complications.

## Data Availability

Not applicable.

## Abbreviations

PPI: Proton-pump inhibitor
CTA: Computed tomography angiography
TIPS: Transjugular intrahepatic portosystemic shunt
